# Complete Genome Sequence of the Antibiotic-Resistant Pseudomonas fluorescens Strain Ant01, from the Rhizosphere of Antarctic Moss

**DOI:** 10.1128/mra.01057-22

**Published:** 2022-12-12

**Authors:** Jisub Hwang, Hyoungseok Lee, Hackwon Do, Sung Gu Lee, Tae-Jin Oh, Jun Hyuck Lee

**Affiliations:** a Research Unit of Cryogenic Novel Material, Korea Polar Research Institute, Incheon, Republic of Korea; b Department of Polar Sciences, University of Science and Technology, Incheon, Republic of Korea; c Division of Life Sciences, Korea Polar Research Institute, Incheon, Republic of Korea; d Department of Life Science and Biochemical Engineering, Graduate School, SunMoon University, Asan, Republic of Korea; University of Arizona

## Abstract

Pseudomonas fluorescens Ant01 was isolated as an antibiotic-resistant strain from the rhizosphere of a moss from Barton Peninsula, King George Island, Antarctica. The assembled genome size is 6,249,144 bp, with 5,616 protein-coding genes, 69 tRNA genes, and 19 rRNA genes.

## ANNOUNCEMENT

Heretofore, numerous Pseudomonas fluorescens strains have been isolated from various environmental sources and their genomes characterized. Among Pseudomonas spp., organisms in the P. fluorescens group are widely known as plant growth-promoting rhizobacteria (1, 2), and several P. fluorescens strains are receiving attention as opportunistic pathogens (3, 4); this implies the danger of an environmental microorganism becoming an opportunistic pathogen, with the necessity to monitor microorganism spread and pathogenicity (5).

P. fluorescens Ant01 was isolated from rhizosphere soil from Antarctic moss (Sanionia uncinata) from Barton Peninsula, King George Island, Antarctica (62°13′11.4″S, 58°46′04.4″W). A sample was collected on 18 January 2018. To prevent inclusion of moss residues, the soil just below the humus layer was spread on a Mueller-Hinton (MH) agar plate containing antibiotics and was incubated at 15°C. Bright yellow colonies were formed on three different antibiotic-containing plates (with 100 μg/mL ampicillin, 30 μg/mL chloramphenicol, and 100 μg/mL streptomycin) after 1 week. A single colony was transferred to fresh MH broth for enrichment of cells. Molecular identification was performed by PCR with the universal primers 27F and 1492R, with the following amplification procedure: denaturation at 95°C for 5 min, followed by 31 cycles of 95°C for 30 s, 57°C for 30 s, and 72°C for 84 s. The PCR product was sequenced with primers 785F and 907R. The results showed 99.93% sequence identity with the type strain P. fluorescens CCM 2115 (GenBank accession number NR_115715.1).

Genomic DNA extraction from the previous broth culture was performed using a Wizard genomic DNA extraction kit (Promega, USA), followed by RNase A treatment and purification according to the kit instructions. A whole-genome sequencing library was prepared using a SMRTbell express template preparation kit (Pacific Biosciences [PacBio], USA). To verify the size of the DNA library, the distribution of template sizes was examined with a 2100 Bioanalyzer (Agilent Technologies, USA) using a DNA 1000 chip. The concentration of the library sample was estimated using a Qubit fluorometer (Qiagen, USA). The manufactured library was sequenced on a PacBio Sequel instrument at Macrogen (Seoul, South Korea), which resulted in a total of 73,401 reads, with an *N*_50_ value of 10,706 bp. Long-read sequencing data were assembled with the Microbial Assembly application in single-molecule real-time (SMRT) Link v10.2 (PacBio, USA). The whole genome was shown to be a single circular contig with a complete level of assembly, with a length of 6,249,144 bp, a G+C content of 59.9%, and a coverage depth of 87.2×. Genes were identified and annotated with the Prokka v1.13 pipeline, which resulted in 5,616 coding genes, 69 tRNA genes, and 19 rRNA genes (6). Default parameters were used for all software unless otherwise specified.

Web-based software, i.e., the Resistance Gene Identifier (RGI) v6.0.0 from the Comprehensive Antibiotic Resistance Database (CARD) v3.2.5, was used for prediction of antibiotic resistance genes (ARGs) (7). The results showed that P. fluorescens strain Ant01 possesses predicted genes that confer resistance to fluoroquinolones, cephalosporins, glycylcyclines, penams, tetracycline, rifamycins, phenicols, disinfecting agents, and antiseptics, with >67% identity. The results are summarized in Table 1.

**TABLE 1:** ARG prediction results using RGI analysis with CARD

No.	Best-hit ARO^*a*^	Drug class	ARG family	Identity (%)
1	Pseudomonas aeruginosa *soxR*	Fluoroquinolones, cephalosporins, glycylcyclines, penams, tetracycline, rifamycins, phenicols, disinfecting agents, antiseptics	ABC antibiotic efflux pump, MFS antibiotic efflux pump, RND antibiotic efflux pump	70.42
2	*adeF*	Fluoroquinolones, tetracycline	RND antibiotic efflux pump	67.17
3	Acinetobacter baumannii *abaQ*	Fluoroquinolones	MFS antibiotic efflux pump	72.49

a ARO, antibiotic resistance ontology; ABC, ATP-binding cassette; MFS, major facilitator superfamily; RND, resistance-nodulation-cell division. Best-hit ARO, drug class, ARG family, and identity are explained at the CARD website (https://card.mcmaster.ca).

### Data availability.

The complete genome sequence of Pseudomonas fluorescens strain Ant01 has been deposited in GenBank under the BioProject accession number PRJNA879781, the BioSample accession number SAMN30814564, and the GenBank accession number CP104408. The raw reads can be accessed under the SRA accession number SRR21601282.
